# Comparative outcomes among inlay grafted incised plate, onlay preputial flap and tubularized incised plate urethroplasty for the repair of distal penile hypospadias with a narrow urethral plate

**DOI:** 10.1007/s00345-023-04690-8

**Published:** 2023-11-10

**Authors:** Mostafa M. Ali, Ahmed Z. Anwar, Mostafa Sh. Mohamed, Ahmed H. Abdelgawad, Mahmoud F. Rohiem, Alayman Hussein, Mohammed G. S. Hasanein

**Affiliations:** 1 Department of Urology, Urology and Nephrology Hospital, Faculty of Medicine, Minia University, Minia, Egypt; 2 Department of Urology, Port Said University Hospital, Port Said University, Port Said, Egypt

**Keywords:** Distal hypospadias, Onlay flap-inlay graft, TIPU, Narrow urethral plate

## Abstract

**Purpose:**

We conducted this study, comparing the outcomes among Transverse Onlay Island Flap, inlay grafted incised plate and our previous records of tubularized incised plate urethroplasty (TIPU) in patients with narrow urethral plates, aiming to determine which method of repair provides a good outcome.

**Methods:**

This hybrid study included two datasets. The first from a prospective randomized study evaluating outcomes of two treatment modalities; Inlay graft and only flap for distal hypospadias with shallow urethral plate with 80 patients (40 patients in each group) included, the second based on our previous records of TIPU in 40 patients with distal primary hypospadias with narrow urethral plate.

**Results:**

The success rate in inlay graft urethroplasty group (*n* = 40) was 87.5%; glandular dehiscence occurred in one case (2.5%), fistulas occurred in 2 cases (5%), and narrow meatus occurred in two cases (5%). Success rate in onlay flap urethroplasty group (*n* = 40) was 82.5%; glandular dehiscence occurred in two cases (5%), fistulas occurred in two cases (5%), and narrow meatus occurred in three cases (7.5%). TIPU group (*n* = 40) had success rate of 62.5%; glandular dehiscence occurred in eight cases (20%), fistulas occurred in five cases (12.5%), and narrow meatus occurred in seven cases (17.5%), with five cases exhibiting both narrow meatus with fistula.

**Conclusion:**

Inlay graft and onlay flap urethroplasty for repair of distal penile hypospadias with narrow urethral plate had higher success rate and fewer complications than traditional TIPU. Moreover, operative time was shorter in TIPU.

## Introduction

In 1994, Snodgrass reported the use of tubularized incised plate urethroplasty (TIPU) with a urethral plate incision that not only widened the plate but also provided a slit-like vertical neourethral meatus, the incised plate healing is still a matter of debate, some authors think that there is complete re-epithelization while others believe that there is granulation tissue formation that later is healed by gradual fibrosis that can lead to several unfavorable outcomes, including meatal and neourethral stenosis, which require regular urethral dilatation [1–3].

Several factors affecting the outcome of hypospadias repair such as meatal location, age at time of repair, presence of chordee, surgical technique, surgeon experience and urethral plate, the urethral plate characteristics that affect the surgical outcome of TIPU has been studied in many series [4–6]. Holland and Smith disclosed a high complication rate in TIPU with the narrow urethral plate (width of less than 8 mm) [4]. Sarhan et al., reported that complications were more common in patients with urethral plate widths less than 8 mm than those with widths greater than 8 mm [5]. Others denied the role of the urethral plate width on the success rate [7, 8].

Many studies have postulated that the augmentation of the narrow urethral plate with a free inlay inner preputial graft (Snodgraft) or Onlay preputial flap provides superior outcomes than conventional TIPU [9, 10].

Hence, we conducted our study comparing the outcomes among inlay grafted incised plate, onlay Island Flap and our previous records of TIPU in patients with a narrow urethral plate, aiming to determine which method of repair provides a good outcome.

## Patients and methods

This hybrid study included two datasets. The first dataset was from a prospective randomised study carried out from January 2016 to March 2021 study included 80 patients; 40 patients in group I (grafted incised plate) and 40 patients in II group (onlay flap) who had primary distal penile hypospadias with a narrow urethral (defined as a pre-incised urethral plate width of less than 8 mm at the widest distance between UP edges [11]; the second dataset was based on our previous records of 40 patients (group III) who underwent TIPU between 2014 to 2016 with a narrow urethral plate. The study carried out at Urology department, Urology and Nephrology hospital, faculty of Medicine, Minia University after obtaining the faculty ethical committee approval with ID: 502/2022.

Redo cases and patients with circumcision, chordee > 30°, patients received testosterone for treatment of small sized penis and a urethral plate more than 8 mm in width were excluded**.** Urethral plate width, meatal location, presence of chordee and operative outcome were assessed. The patients’ parents signed a detailed written consent form with a description of the surgical techniques and the expected postoperative complications.

### Operative techniques

All operations were performed by two authors. The operation began with a dorsal circumferential subcoronal incision, which was extended ventrally to 2 mm below the hypospadiac urethral meatus. Subsequently, the penis was degloved, and the urethral plate width was measured. The glanular wings were prepared. A deep midline incision was made along the urethral plate and extended from the hypospadiac meatus to the tip of the glans. Each patient’s randomised group assignment was provided in an envelope, which was opened immediately following the glanular wings formation and urethral plate incision.

### Inlay graft [12]

**Fig. 1:**
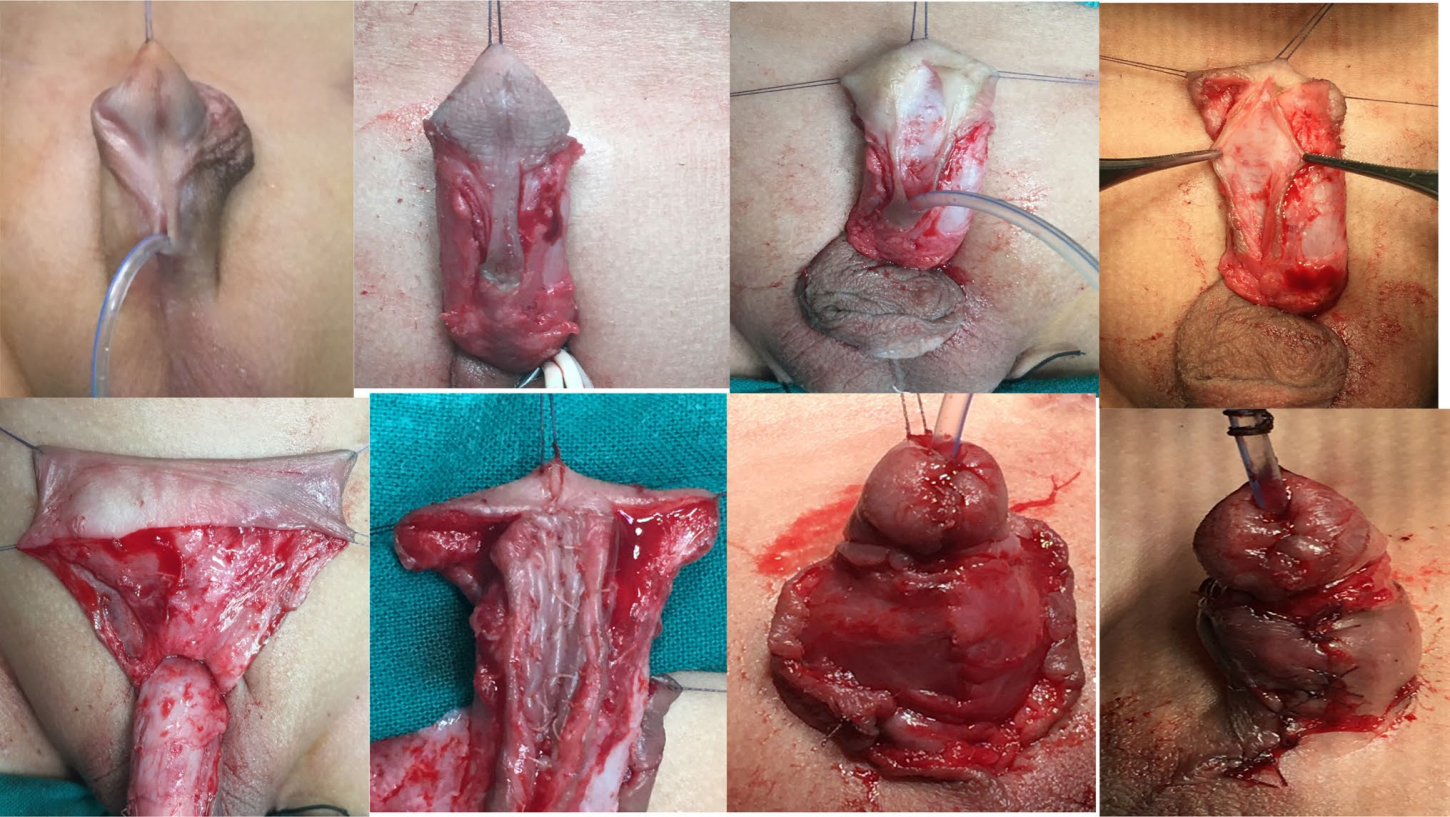
The graft was outlined, harvested, and defatted from the inner preputial skin; it was then placed over the incised urethral plate and fixed to its edges from the original meatus to the tip of the glans at the mucocutaneous junction

The graft was outlined, harvested, and defatted from the inner preputial skin; it was then placed over the incised urethral plate and fixed to its edges from the original meatus to the tip of the glans at the mucocutaneous junction with 7/0 Vicryl sutures. The graft was quilted to the underlying corpora in the incised plate defect. Urethroplasty was performed in two layers; continuous subcuticular sutures (7/0 Vicryl) were applied in the first layer, and interrupted sutures (7/0 Vicryl) were used in the second. A second layer of coverage with a dorsal dartos flap was applied in all patients. Glanular approximation was performed with 6/0 Vicryl in the two layers. Finally, skin closure was performed. A suitable urethral stent was retained for 5–7 days (Fig. 1).

### Onlay flap [13]

**Fig. 2:**
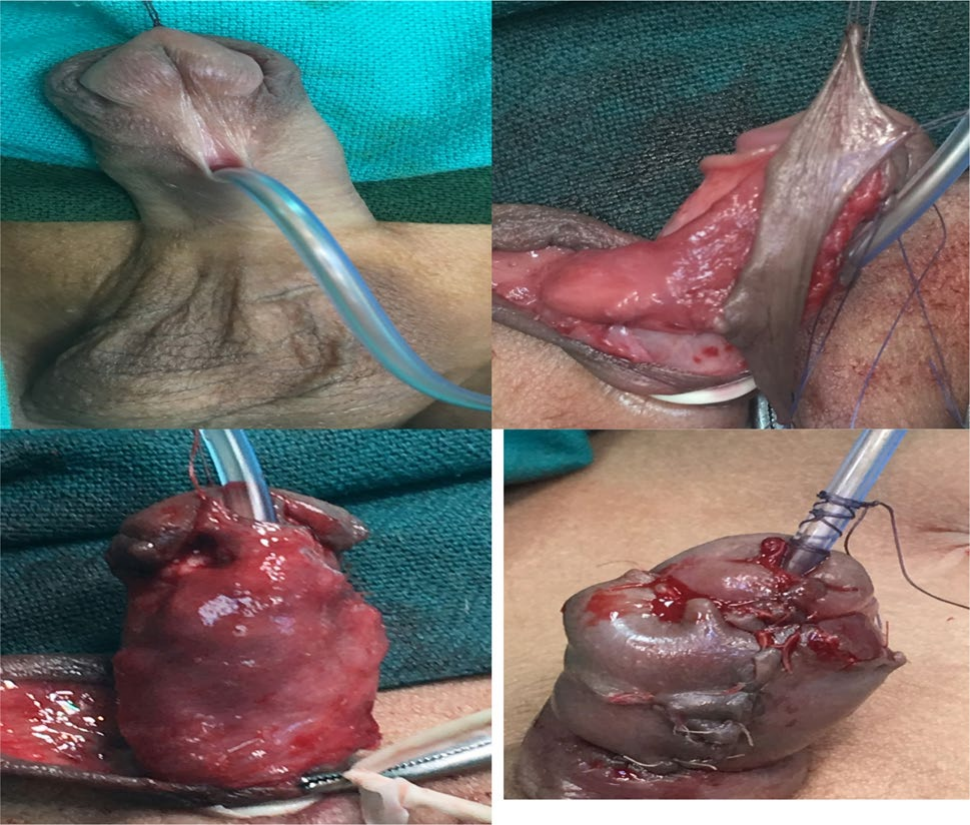
A transverse inner preputial flap was outlined with a minimum width of 8 mm, and the length was adjusted according to the distance between the hypospadiac meatus and the tip of the glans. The flap was dissected with the dartos pedicle, rotated ventrally, and sutured to the urethral plate edges

A transverse inner preputial flap was outlined with a minimum width of 8 mm, and the length was adjusted according to the distance between the hypospadiac meatus and the tip of the glans. The flap was dissected with the dartos pedicle, rotated ventrally, and sutured to the urethral plate edges with 7/0 Vicryl sutures over a urethral catheter. The dartos pedicle was utilised as a second layer to cover the suture lines. Finally, glanular and skin closure was performed as previously described. The urethral catheter was retained for 8–10 days (Fig. 2).

### Tubularised incised plate urethroplasty as described by Snodgrass [1]

**Fig. 3:**
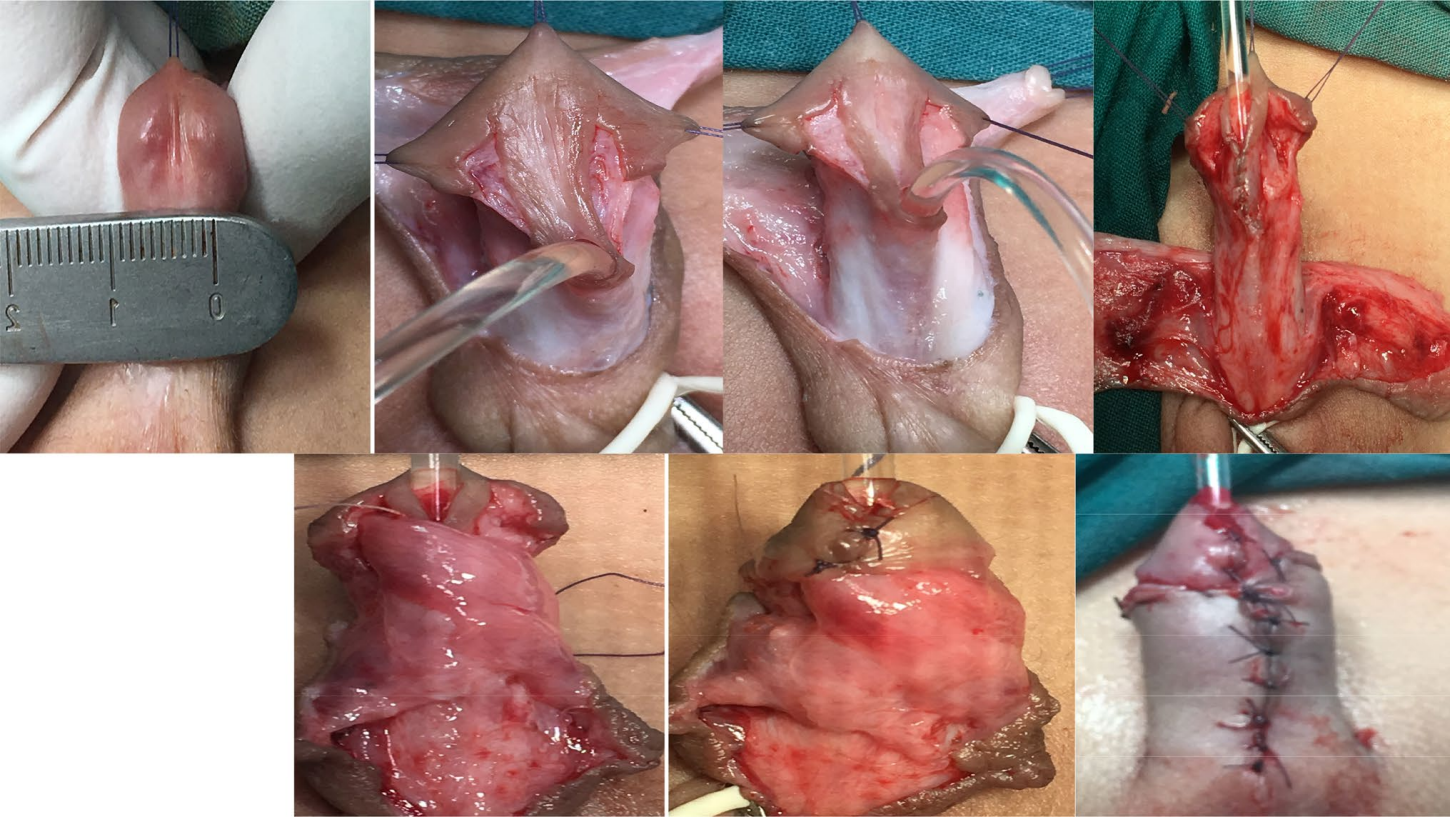
TIPU for narrow urethral plate. Bilateral longitudinal glanular incision that laterally dissected. A deep midline incision was made along the urethral plate and extended from the hypospadiac meatus to the tip of the glans was performed. A subcuticular closure of the neourethra was achieved with 7/0 Vicryl sutures over a urethral catheter. A second covering layer from the dorsal dartos was applied. Finally, glanular and skin closure

A subcuticular closure of the neourethra was achieved with 7/0 Vicryl sutures over a urethral catheter. A second covering layer from the dorsal dartos was applied. Finally, glanular and skin closure was performed as previously described. Patients were discharged with an indwelling catheter measuring 6-8F, which was removed on days 5–7 following surgery (Fig. 3).

A Coban 3 M™ self-adherent dressing was used for all patients. The dressing was retained for 5 days and then removed. The surgical outcome was classified as either a “success” or a “complication.”. Success was defined as a straight penis with a conically shaped glans and a slit-like meatus located at the glans tip, along with a forward-directed single stream with an adequate caliber during the follow up period.

The patients were followed up with regular visits 1, 3, 6, and 12 months after operation. At each visit, the occurrence of complications was assessed, such as glans dehiscence, fistula formation, meatal stenosis, and diverticulum formation.

### Sample size calculation

Before the study, the number of patients required in each group was determined after a power calculation according to data obtained from previous studies. In those studies, the complication rate in group I was 8% [14] and in group II was 33% [15]. a sample size of 40 patients in each group was determined to provide 80% power at the level of 0.05 significance using G Power 3.1 9.2 software. We used the same number for group III.

### Statistical analysis

The data were analysed by Statistical Package for Social Sciences program (SPSS) software version 20. Descriptive statistics for numerical variables are presented as the mean and standard deviation, and those for qualitative variables are presented as numbers and percentages. One-way analysis of variance (ANOVA) was used to assess differences in quantitative variables between the three groups. The Chi-square test and Fisher’s exact were used to assess differences in qualitative variables between the three groups. A *p*-value of <0.05 was considered significant.

## Results

**Fig. 4:**
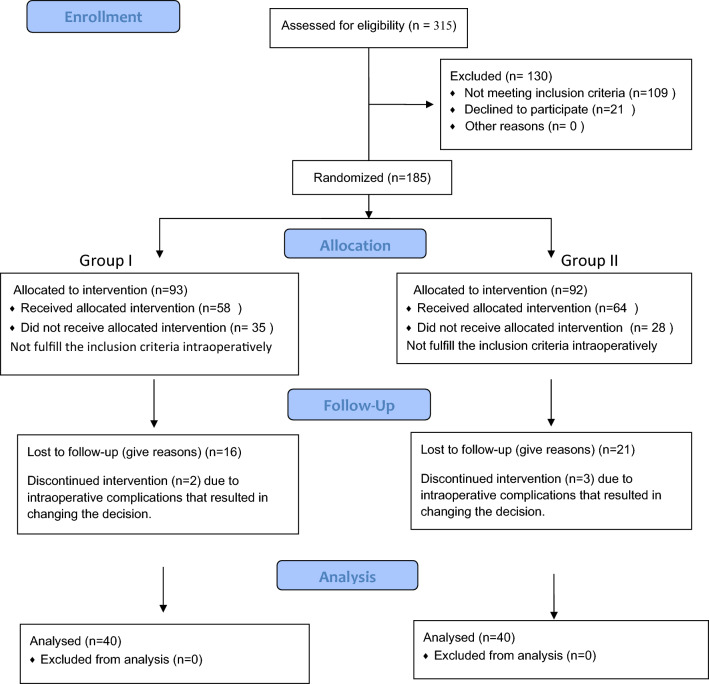
Consort 2010 flow diagram

Of the 315 hypospadias cases at our institution during the study period, 80 met our inclusion criteria (Fig. 4). All groups were comparable regarding age, meatal location and urethral plate width (Table 1). The urethral plate width did not differ significantly between the groups (*p* = 0.160), and all patients had a urethral plate width of less than 8 mm. Cases with chordee were less than 30° and most of them were corrected after penile degloving; Nesbit dorsal plication was applied in four, three, and five cases in groups I, II, and III, respectively. The mean operative time was 98 ± 14 min in group I (graft preparation and implantation: 32–50 min; mean: 38 ± 9 min), 86 ± 25 min in group II, and 71 ± 22 min in group III, with a significant difference between group III and the other groups. The minimal follow-up period was 18 months.

**Table 1:** Patients demographics and outcome in the three studied groups

Patients’ demographics	Group I (*n* = 40)	Group II (*n* = 40)	Group III (*n* = 40)	*p* Value		
Patient age (month)	10–49	15–45	11–52	0.319		
	31 ± 18	28 ± 17	34 ± 18	Ӏ and II	IӀ and III	Ӏ and III
				0.449	0.132	0.449
Meatal location						
Coronal	12 (30%)	12 (30%)	11 (27.5%)	0.971	Ӏ and II (0.965)	
Sub coronal	13 (32.5%)	14 (35%)	16 (40%)		II and III (0.899)	
Distal penile	15 (37.5%)	14 (35%)	13 (32.5%)		Ӏ and III (0.780)	
Urethral plate width				0.412		
Range	4–7	5–8	5–7	Ӏ and II	II and III	Ӏ and III
Mean ± SD	5.6 ± 1.2	6 ± 1.1	5.8 ± 0.8	0.378	0.753	0.811
Chordee	9	11	10	0.875		
				Ӏ and II	II and III	Ӏ and III
				0.606	0.263	0.799

Out come	Group Ӏ (*n* = 40)	Group II (*n* = 40)	Group III (*n* = 40)	*p*-Value		
				Ӏ and II	II and III	Ӏ and III
Operative time (min.) Range Mean ± SD	81–112 98 ± 14	76–111 86 ± 25	60–93 71 ± 22	< 0.01		
				0.011*	0.0001*	0.0001*
Success	35 (87.5%)	33 (82.5%)	25 (62.5%)	0.018*		
				0.531	0.045*	0.010*
Fistula	2 (5%)	2 (5%)	5 (7.5–12.5%)	0.339		
				1	0.432	0.432
Narrow meatus	2 (5%)	3 (7.5%)	7 (17.5%)	0.143		
				0.644	0.311	0.154
Glans dehiscence	1 (2.5%)	2 (5%)	8 (20%)	0.014*		
				0.556	0.043*	0.029*
Diverticulum	0	0	0			

The overall complication rate in the inlay graft urethroplasty group was (15/40); glandular dehiscence occurred in only one case (2.5%), fistulas occurred in two cases (5%), and narrow meatus occurred in two cases (5%). While in the onlay flap urethroplasty group, complications were occurred in seven cases; glandular dehiscence occurred in two cases (5%), fistulas occurred in two cases (5%), and narrow meatus occurred in three cases (7.5%), The TIPU group (*n* = 40) had a success rate of 62.5% (25/40 cases); glandular dehiscence occurred in eight cases (20%), fistulas occurred in five cases (12.5%), and narrow meatus occurred in 7 cases (17.5%), with five cases exhibiting both narrow meatus with fistula (Table 1). No case within the three groups developed urethral diverticulum.

Regarding the success rate and glandular dehiscence, a statistically significant difference was noted between group III and both groups I and II. On the other hand, cases with urethral fistula and meatal stenosis were higher in group III in comparison with group I and II but without a statistically significant difference (Table 1).

## Discussion

TIPU repair for distal and mid-penile hypospadias has gained widespread acceptance because it is versatile, has a low complication rate, and reliably results in a vertically oriented meatus. Various factors affect the success rate of hypospadias repair, among which is the urethral plate [6, 13]. Many authors utilized the inner prepuce for augmentation of the narrow urethral plate, Kolon and Gonzales reported a technique for one-stage urethroplasty utilising a free inner preputial graft that was inlaid within the incised plate; this method resulted in an increased urethral plate width and reduced the risk of neourethral complications in cases with flat and narrow urethral plates. [12]. On the other hand, others utilized the inner prepuce as onlay island flap [13].

Tariq et al. concluded that the urethral plate quality plays an important role as an independent factor which can affect the postoperative results of hypospadias repair, and that the currently used methods for description of urethral plate quality are highly subjective and lacking generalizability [16].

Many studies have compared inlay graft urethroplasty, onlay preputial flap urethroplasty, and TIPU in the repair of distal hypospadias with narrow urethral plate separately, up to our knowledge our study is the only comparative study among the three methods of narrow urethral plate augmentation.

The operative time was 98 ± 14 min in inlay graft group, 86 ± 25 min in the only flap group, and 71 ± 22 min in TIPU group, the operative time was significantly increased in the inlay graft and only flap group in comparison to the conventional TIPU. In agreement with our results, Helmy et al. and Omran et al., reported that the operative time was longer for the patients who underwent the Snodgraft procedure and onlay flap urethroplasty [17, 18].

In our study, in the inlay graft urethroplasty group, the overall complications were occurred in 12.5% of the cases, fistula in two cases (5%), glanular dehiscence occurred in one case (2.5%), and narrow meatus occurred in two cases (5%). While, in the onlay flap urethroplasty group (*n* = 40), fistulas occurred in two cases (5%), glanular dehiscence occurred in two cases (5%), and narrow meatus in three cases (7.5%), with no cases with diverticulum or flap loss. Similarly, the study by Omran et al. (2021) reported only one case of fistula and glandular dehiscence in 23 cases (5.3%) with narrow urethral plate augmented by inlay graft in anterior and middle hypospadias repair, while the onlay flap urethroplasty group (20 patients) reported urethral fistulas in five patients (25%), glandular dehiscence in two patients (10%), diverticulum in one patient (5%), and flap loss in one patient (5%) [18], while in our study we didn`t report any case with urethral diverticulum in the only flap group. Also, Seleim et al. reported two cases of fistulas in 81 patients with narrow urethral plates corrected by inlay graft (2.5%) [19].

In our study the success rate in TIPU was significantly low (*p* < 0.05) in comparison to inlay graft group, also higher number of complications; fistulas occurred in 5/40 (12.5%), glandular dehiscence occurred in eight cases (20%), and narrow meatus occurred in 7/40 (17.5%); two cases showed both narrow meatus with fistula, nearly similar, Shimitokahara et al., performed 100 operations and reported that complications were significantly less frequent in the dorsal inlay graft group (4 cases) than in the TIPU group (15 cases, *p* < 0.05); in the dorsal inlay graft group, no patients exhibited meatal stenosis, only one case exhibited neourethral stenosis without distal diverticulum, and three cases exhibited urethrocutaneous fistulas. By contrast, in the TIPU group, two patients had meatal stenosis, six patients had neourethral stenosis with or without distal diverticulum, and seven patients had urethrocutaneous fistulas. The authors concluded that the Snodgraft technique decreased urethral stenosis and fistula formation [14]. However, this was a retrospective study that included all types of hypospadias and did not measure the urethral plate width.

Conversely, Helmy et al., reported that of 30 patients who underwent operations using the dorsal inlay graft technique, only two showed glandular dehiscence. The authors found no significant differences between patients who received dorsal inlay graft and those who underwent TIPU in terms of the success rate (96.7% vs 93.3% respectively), cosmetic outcome, and flow rate (including *Q*-max and voided time). However, the dorsal inlay graft group showed a significantly longer operation time (*p* = 0.005) [17]. However, this study excluded cases with a urethral plate width of < 8 mm, and the sample size was relatively small.

Mouravas et al. [9] performed a comparative analysis between dorsal inlay graft urethroplasty and conventional TIPU in 47 patients. Dorsal inlay graft urethroplasty resulted in one fistula and one glandular dehiscence (4.2% for each), whereas in the conventional TIPU group, complications occurred in seven cases (30.4%), glandular dehiscence occurred in one case (4.2%), urethra stenosis occurred in six cases (26.1%), and fistulas occurred in two cases (8.4%). Therefore, the authors recommended dorsal inlay graft urethroplasty as the procedure of choice for patients undergoing primary hypospadias repair.

In 2022, El-Helaly et al., compared the Snodgrass technique (33 cases) and transverse preputial onlay flap (33 cases) in distal hypospadias repair. Postoperative urinary fistulas occurred in 15.2% of cases in the Snodgrass group, whereas no cases of fistulas occurred in the transverse preputial onlay flap group (*p* = 0.05). The vertical slit appearance of the meatus was better in the Snodgrass group (54.5%) than it was in the transverse preputial onlay flap group (24.2%, *p* = 0.023). The mean operation time was significantly higher in the transverse preputial onlay flap group (123.1 ± 6.8 min) than in the Snodgrass group (93.73 ± 3.9 min, *p* < 0.001) [20].

Few studies have investigated TIPU in patients with narrow urethral plates. One study performed by Holand and Smith [21] reported an increased rate of complications in patients with a urethral plate width of less than 8 mm; these included urethral fistula (55%) and meatal stenosis (18%). However, a 2020 study by Eldeeb et al. reported that in 30 patients with narrow urethral plates who received TIPU, only one patient had meatal stenosis and one had a distal penile fistula [22]. Nguyen and Snodgrass [23] measured the urethral plate width in 48 patients; 30 patients had a urethral plate width of less than 8 mm, of whom only one developed a urethral fistula and none exhibited meatal stenosis. This suggested that the width of the urethral plate did not affect the short-term outcomes (within 8 months) of TIPU; however, this study did not assess the narrow urethral plate width as a univariant affecting the success of TIPU. By contrast, Sarhan et al. [5] reported that of 80 patients who underwent the operation, 39 had a urethral plate width of less than 8 mm, and these patients had a significantly higher complication rate than patients with a wide urethral plate.

Limitation of the study included; retrospective nature of the TIPU group, the study should be run on large number of patients allowing more stratification of the urethral plate width; allowing comparison within the groups as regarding the success rate in relation to urethral pate width, measurement of the incised plate should be put in mind in further studies and absence of glandular size measurement and application of GMS score.

## Conclusions

Our data indicated that inlay graft and only flap urethroplasty (inlay graft urethroplasty or onlay preputial flap urethroplasty) for the repair of distal penile hypospadias with narrow urethral plate had a higher success rate and fewer complications than traditional TIPU in this group of patients. Moreover, the operation time was shorter in TIPU in comparison to other methods of repair.
